# Translocated LPS Might Cause Endotoxin Tolerance in Circulating Monocytes of Cystic Fibrosis Patients

**DOI:** 10.1371/journal.pone.0029577

**Published:** 2011-12-28

**Authors:** Rosa del Campo, Eriel Martínez, Carlos del Fresno, Raquel Alenda, Vanesa Gómez-Piña, Irene Fernández-Ruíz, María Siliceo, Teresa Jurado, Victor Toledano, Francisco Arnalich, Francisco García-Río, Eduardo López-Collazo

**Affiliations:** 1 Servicio de Microbiología and CIBER en Epidemiología y Salud Pública (CIBERESP), Instituto Ramón y Cajal de Investigación Sanitaria (IRYCIS), Hospital Universitario Ramón y Cajal, Madrid, Spain; 2 EMPIREO Research S.L., Madrid, Spain; 3 Laboratory of Tumor Immunology, IdiPAZ, ‘La Paz’ Hospital, Madrid, Spain; 4 Department of Inmunology, University Hospital Ramon y Cajal and IRYCIS, Madrid, Spain; 5 Service of Internal Medicine, ‘La Paz’ Hospital, Madrid, Spain; 6 Service of Respiratory Diseases, ‘La Paz’ Hospital, Madrid, Spain; Hospital Vall d'Hebron, Spain

## Abstract

Cystic Fibrosis (CF) is an inherited pleiotropic disease that results from abnormalities in the gene codes of a chloride channel. The lungs of CF patients are chronically infected by several pathogens but bacteraemia have rarely been reported in this pathology. Besides that, circulating monocytes in CF patients exhibit a patent Endotoxin Tolerance (ET) state since they show a significant reduction of the inflammatory response to bacterial stimulus. Despite a previous description of this phenomenon, the direct cause of ET in CF patients remains unknown. In this study we have researched the possible role of microbial/endotoxin translocation from a localized infection to the bloodstream as a potential cause of ET induction in CF patients. Plasma analysis of fourteen CF patients revealed high levels of LPS compared to healthy volunteers and patients who suffer from Chronic Obstructive Pulmonary Disease. Experiments *in vitro* showed that endotoxin concentrations found in plasma of CF patients were enough to induce an ET phenotype in monocytes from healthy controls. In agreement with clinical data, we failed to detect bacterial DNA in CF plasma. Our results suggest that soluble endotoxin present in bloodstream of CF patients causes endotoxin tolerance in their circulating monocytes.

## Introduction

The incidence of Endotoxin Tolerance (ET), defined as a state of reduced responsiveness to an endotoxin challenge after a primary bacterial insult [1], has been reported in the settings of several diseases including sepsis, trauma, and coronary syndromes [2]–[5].

Cystic Fibrosis (CF) is a complex disease that affects essentially all exocrine epithelia [6]. CF results from abnormalities in the gene that codes for the chloride channel termed CF Transmembrane Conductance Regulator (CFTR), which belongs to the extended family of ATP-binding cassette (ABC) transporter ATPases [6]. This transmembrane glycoprotein is expressed in some epithelia, and controls chloride flux across cell surfaces. In addition, it down-regulates transepithelial sodium transport, regulates calcium-activated chloride channels and potassium channels, and may also serve important functions in exocytosis. Some clinical features of CF include injuries of primary organs (pancreas, sinus, liver, intestine and exocrine pancreas) and secondary complications such as malnutrition and diabetes. However, morbidity and mortality of CF patients are usually the result of chronic lower airway bacterial infections and inflammation of the lungs. Repeated episodes of polymicrobial infection in these patients cause a progressive deterioration of lung tissue, a decline in pulmonary function and, ultimately, respiratory failure and death in 90% of CF patients. In this regard, the observed high frequency of pathogen colonization in these patients points to a significant deficiency of their innate immune system [6], [7].

A number of studies conducted so far have focused on local and resident cells (e.g. lung epithelial cells and neutrophils), and most of them described a defective secretion of pro-inflammatory cytokines [8]. Our previous findings revealed a patent ET status in circulating monocytes (M
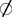
s) isolated from CF patients [9], [10]. These cells are unable to mount a standard inflammatory response after *ex vivo* endotoxin challenge. Besides that, we also have noticed other main features of ET status in their M
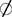
∅s (e.g. high phagocytosis ability and poor antigen presentation) [9], [10]. Additionally, a low expression of TREM-1 at cell surface has been detected in circulating CF-M
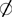
s [9]. This orphan receptor magnifies the inflammation after TLR activation in myeloid cells and is implicated in a number of inflammatory pathologies [11]. The low levels of TREM-1 expression in circulating CF M
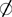
s partially justify the non-responsiveness state in CF patients.Nevertheless, the answer to the question “Why are circulating cells from CF patients tolerant?” is largely unknown.

The translocations of microorganisms and/or microbial products have been previously described in other pathologies, such as HIV, Inflammatory Bowel Disease and pancreatitis [12]–[14]. Microbial translocation also occurs after damage to the gastrointestinal tract (e.g. after cholecystectomy) resulting in systemic immune deregulation [15], [16]. The quantity of LPS, the major component of the outer membrane of Gram-negative bacteria, is frequently associated with the degree of bacterial translocation in several diseases [13], [17], [18]. In the particular case of CF pathology, bacteremia has been rarely described and the levels of circulating soluble LPS have yet to be determined [19].

The goal of the present study was to analyze a possible role of circulating soluble LPS on the ET status in CF patients. To accomplish this we first corroborated the ET status in a cohort of fourteen CF patients. Second, we quantified the plasma levels of LPS in these patients and the data were compared to healthy controls and patients who suffer from Chronic Obstructive Pulmonary Disease (COPD). We also evaluated the presence of bacterial DNA or viable cells. Finally, we determined if LPS concentrations found in CF plasma were enough to induce ET in human monocytes *in vitro*.

## Results

### Circulating monocytes from CF patients exhibit an Endotoxin Tolerant phenotype

**Figure 1:**
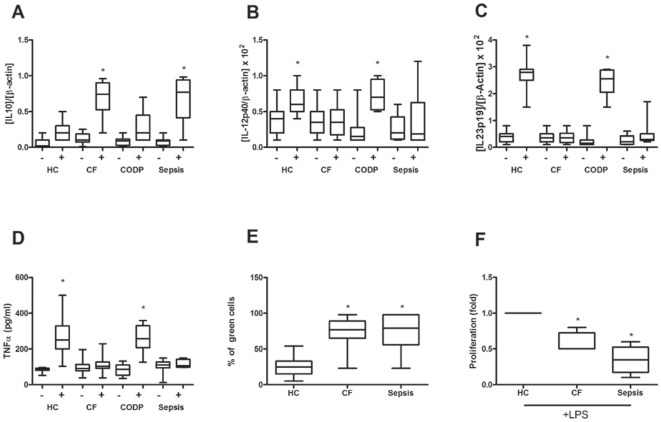
Circulating monocytes from CF patients exhibit an Endotoxin Tolerant phenotype. Monocytes from healthy controls (HC, n = 11), CF patients (CF, n = 14), COPD patients (n = 8) and sepsis patients (n = 8) were isolated from circulation and cultured in the presence or not of 10 ng/ml LPS for 3 h. Then, cells were harvested and mRNA levels of IL10 (*A*), IL12p40 (*B*) and IL23p19 (*C*) were determined by real-time Q-PCR. The ratios [gene]/[β-actin] are depicted. *, *p*<0.01, LPS vs. none. (*D*) Concentrations of TNFα were determined by ELISA in supernatants of cultures after 24 h of stimulation with 10 ng/ml LPS. *, *p*<0.05 LPS *vs.* none. (*E*) Cultures of circulating M
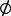
s from healthy controls (HC, n = 11), CF (n = 14) and sepsis (n = 8) patients were exposed to GFP-labelled *E. coli* DH5α bacteria for 1 h according to the protocol described in *Materials and Methods*. Next, adherent cells were harvested and cell internalization was analyzed by flow cytometry. Percentage of green-positive cells is given, *, p<0,05 *vs.* HC. (*F*) Heterologous human lymphocytes isolated from healthy volunteers were labelled with the membrane stain PKH2 green fluorescent cell linker kit. Following *ex vivo* LPS stimulation of monocytes/macrophages isolated from healthy controls (HC, n = 11), CF (n = 14) and sepsis (n = 8) patients for 24 h, stained lymphocytes were added to the plates as responder cells in a relation of 1∶5. After three days, non adherent cells were harvested, and lymphocyte proliferation was assessed by flow cytometry as loss of green fluorescence intensity in the CD3+ gate. The fold induction is shown (*, *p*<0.05 vs HC).

According to our previous reports [9], [10] circulating M
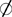
s isolated from CF patients fail to mount an appropriate inflammatory response in the presence of Gram-negative endotoxin. The reported “ET-signature” (IL10^high^/IL12^low^/IL23^low^) was corroborated in this new cohort of CF patients (n = 14) (**Fig. 1A–1C**) as well as a low TNFα production after *ex vivo* LPS challenge (**Fig. 1D**). However, endotoxin tolerance was detected neither in healthy controls (n = 11) nor in patients suffering from the respiratory disease COPD (n = 8). A group of septic patients used as positive control was included in our study (n = 8). Additionally, cells from CF as well as sepsis patients showed high phagocytosis ability and impaired antigen presentation (**Fig. 1E and 1F**), both are main features of an endotoxin tolerant phenotype [1], [10]. Note that a set of other cytokines and chemokines (IL6, CCL3, CCL4, CCL20 and CCL22) were checked to verify the ET status of CF patients (data not shown).

### Significant levels of bacterial endotoxin are detected in the plasma of CF patients

**Figure 2:**
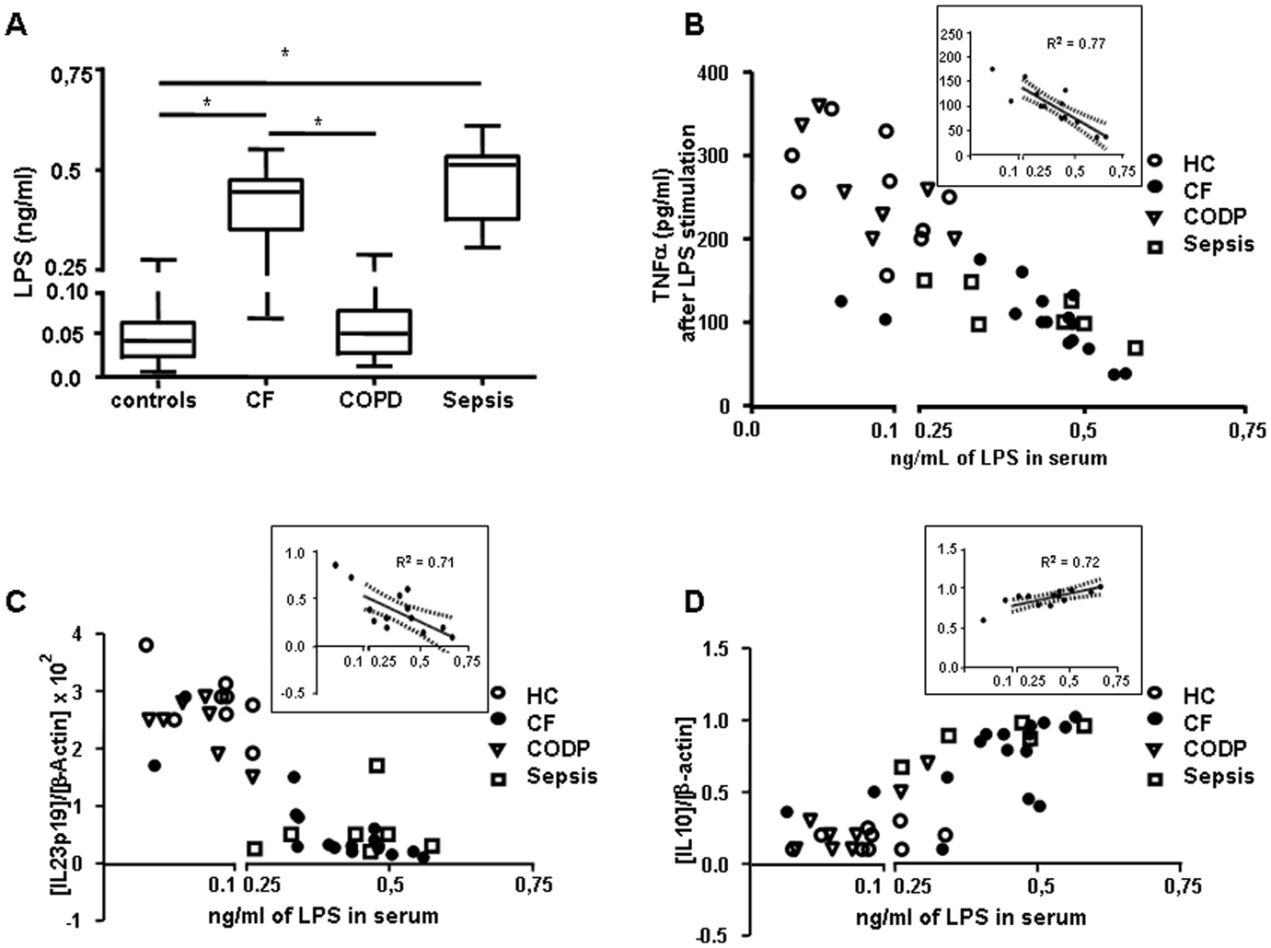
Circulating endotoxin levels correlates with the refractory state in CF patients. (*A*) Levels of LPS were tested by LAL kit endpoint-QCL1000 (see Materials and Methods) in plasma from controls (HC, n = 11), CF (n = 14), COPD (n = 8) and sepsis (n = 8) patients (*, *p*<0.01). Circulating monocytes from healthy controls (HC, n = 11), CF patients (CF, n = 14), COPD patients (n = 8) and sepsis patients (n = 8) were isolated and cultured in presence of 10 ng/mL of LPS for 24 h (*B*), and 3 h (*C* and *D*). Next, levels of TNFα production were analysed by ELISA in the supernatant of cultures (*B*) or cells were harvested and mRNA of IL23p19 and IL10 were quantified by real time Q-PCR (*C* and *D*), (*B–D, insets*). Linear regression analysis was applied in each case to obtain best linear equation with CF only (R^2^ is given).

Having established that CF patients suffer from an ET status, no strong evidence has reported the cause of this phenomenon. In the case of sepsis the presence of circulating traces of endotoxin or viable pathogen itself provides an explanation for their endotoxin tolerance. However, in general terms CF patients suffer a local rather than a systemic infection [20]. Others authors have reported noteworthy levels of microbial products in the circulation of HIV patients, a fact that correlates to systemic immune deregulation in chronic HIV infection [17], [21]–[23]. In those patients detected endotoxins probably derived from the gastrointestinal tract and have an impact on the innate immune control. These reports prompted us to study the presence of LPS in the plasma of CF patients. As shown in **figure 2A**, significant levels of endotoxin were detected in CF plasma (∼0.3 ng/ml). In contrast, low quantities of endotoxin were reported in both healthy controls and COPD patients (∼0.05 ng/ml). These data suggested a role for endotoxin in the generation of an ET status in these patients. In fact, in a global analysis including all groups, we found that endotoxin concentrations inversely correlated to the inflammatory response after an *ex vivo* LPS challenge (**Fig. 2B–2C**). Those M
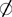
s from patients with high levels of LPS (CF and sepsis) were unable to produce significant TNFα quantities after *ex vivo* LPS challenge (**Fig. 2B**). Similar results were obtained when the expression of IL23p19 mRNA was analysed (**Fig. 2C**). In contrast, LPS concentration correlates with high IL10 mRNA expression after *ex vivo* endotoxin stimulation (**Fig. 2D**). In addition, a significant correlation (*R^2^*∼0.7) was obtained when CF patients were analyzed (**Fig. 2B–2D** **, insets**). Note that two CF-patients were not included in the analysis (insets) because their LPS concentration was very low (<0.1 ng/mL) in comparison to the rest of the patients enrolled. However, their innate response after *ex vivo* LPS-challenge were consistent the observed tendency.

### A metagenonic analysis of CF plasma suggests the absence of microbes in the circulation of these patients

**Figure 3:**
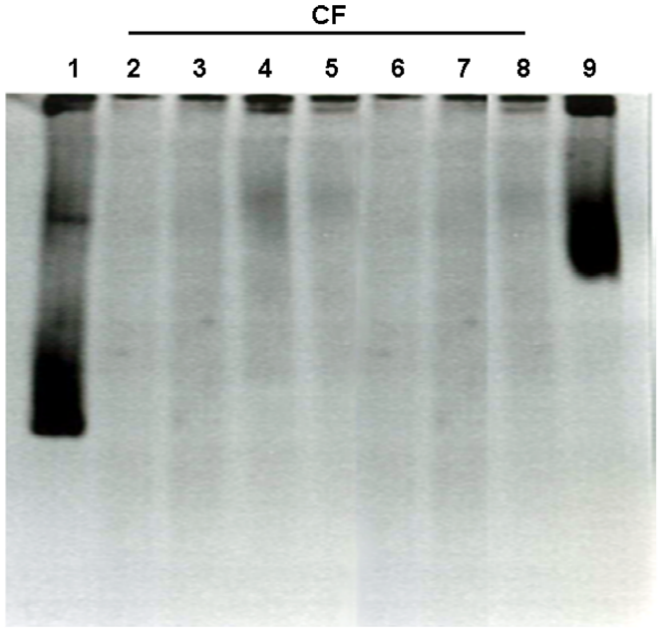
Serum from CF patients does not exhibit bacterial DNA. A metagenomic assay was performed using the plasma from seven CF patients randomly selected. DNA was extracted from 200 µl of plasma and analyzed following the protocol described in Material and Methods. Positive amplicons were visualized on Acrylamide DGGE gels stained with ethidium bromide. Line 1 *Pseudomonas aeruginosa* PAO-1 strain, lines 2-8 CF serum samples and line 9 methicillin-resistant *Staphylococcus aureus* ATCC 33591 strain.

The detected levels of LPS in plasma of CF patients suggested the presence of microbes in this fluid. Similarly to the case of HIV patients, the source of plasma endotoxin could be bacteria translocation from the gastrointestinal tract [22], [23]. In addition, due to chronic airways colonization that these patients suffer, their lungs could be an alternative source of microbial translocation. To test this hypothesis we studied the putative presence of bacteria in CF plasma using a universal 16S rRNA PCR (seeMaterial and Methods). Consistent negative PCR results were obtained for the CF plasma since no bands were visualized in an Acrylamide Denaturing Gradient Gel Electrophoresis (**Fig. 3**). In addition, negative standard blood cultures were observed in duplicate fresh plasmas from these patients (data not shown).

### Sera from Cystic Fibrosis patients induce a patent down regulation of the inflammatory response in healthy control M
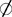
s

**Figure 4:**
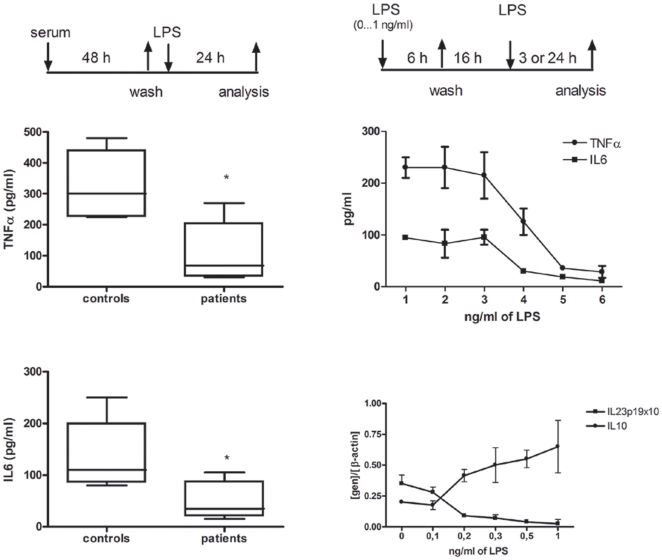
Concentrations of LPS detected in CF patients induce ET in healthy control M
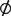
s. (*A*) Schematic representation of the employed experimental design. Monocytes, isolated from healthy controls, were cultured in presence of sera from CF patients (n = 14) and controls (n = 5) for 48 hours. Then, cultures were washed and fresh complete medium and LPS (10 ng/ml) added for 24 h. Next, TNFα (*B*) and IL6 (*C*) production were analyzed by ELISA in the supernatants. (*D*) Schematic representation of the employed endotoxin tolerance model (see Materials and Methods and ref. [10]). Cultures of monocytes, isolated from healthy controls, were pretreated with indicated LPS doses for 6 h. Then, cultures were washed and kept in complete medium for 16 h. After this period of recovery, the cultures were re-challenged with 10 ng/mL of LPS for 24 h (*E*) or 3 h (*F*). Next, TNFα and IL6 (*E*) production were analyzed by ELISA in the supernatants of the cultures and cells were harvested and mRNA of IL10 and IL23p19 were quantified by real time Q-PCR (*F*).

In order to demonstrate that levels of endotoxin detected in plasma from CF patients could be a cause of the observed ET, we cultured human M
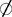
s isolated from healthy controls in presence of supplemental sera from CF patients (50% of total volume). After 48 hours, dishes were washed twice and fresh medium was added. Then, cells were challenged with LPS for 24 hours and the production of TNFα and IL6 was evaluated at the supernatant of each culture (see the experimental design in **figure 4A**). In those cultures “pre-treated” with sera from CF patients but no with controls both, TNFα and IL6 exhibited a marked down regulation of its expression, after LPS challenge (**Fig. 4B and 4C**). Moreover, we have also proven that LPS doses found in CF sera induce ET in healthy monocytes. To test that M
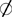
s were pre-stimulated with different doses of LPS, including those detected in CF patients, for six hours. Then, cells were washed and kept in completed medium for 16 hours. Next, a standard LPS dose (10 ng/ml) was added and production of TNFα and IL6 were evaluated 24 hours after in the supernants of these cultures (see the scheme in **figure 4 D**). Note that this experimental design was previously established by us as a solid human model for endotoxin tolerance [1], [10]. Data shown in **figure 4E and 4F** demonstrate that the endotoxin concentrations detected in the plasma of CF patients are sufficient to induce an ET state in human circulating monocytes (∼0.3 ng/ml, **figure 2A**). In this way, we noticed a down regulation of both TNFα and IL6 generation when cells were re-challenged with a standard dose of LPS. Additionally, IL23p19 mRNA expression is also down regulated and IL10mRNA shows a patent increase. Others markers of ET phenotype were also checked and showed a tolerant behavior (data not shown).

## Discussion

Endotoxins are the most potent microbial mediator implicated in the pathogenesis of sepsis and septic shock [24]. In particular LPS, a lipopolysaccharide located on outer membrane of Gram-negative bacteria, has been postulated as the major immunogenic factor of this kind of bacteria. It is well known that small amounts of endotoxin may be released in a soluble form during multiplication or bacterial lyses mediated by complement, lysozyme, phagocytic digestion or antibiotic treatment [25]. A sudden release of large quantities of LPS into the bloodstream starts the generation of a potentially lethal array of inflammatory mediators in circulation. Conversely, a systematic o repetitive release of low levels of LPS into the bloodstream could generate an effect in mononuclear cells known as endotoxin tolerance [1]. Previous research revealed a patent endotoxin tolerance state in circulating monocytes from CF patients. Two studies on two different cohorts of CF patients demonstrated that their peripheral M
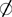
s share all the determinant features of ET, including the IL pattern of expression (IL10^high^/IL12^low^/IL23^low^), high phagocytosis ability and impaired antigen presentation [9], [10].

Herein we have analysed a possible role of circulating cell-free LPS in ET induction in fourteen CF patients. We report significant higher concentrations of LPS in CF plasma than in healthy controls and COPD patients. The ET status in our cohort of CF patients was corroborated by monocyte unresponsiveness to LPS exposure. As we and others previously demonstrated, the observed refractory state in CF is not secondary to impaired LPS recognition by circulating CF-M
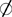
s. The cell surface molecules TLR4 and CD14, together with the MD2 expression, are not affected in these cells [9], [26]. Therefore, we postulate that the ET status in CF patients is due to the presence of circulating endotoxin. In order to prove this hypothesis, we pretreated control human monocytes with a range of LPS doses including the concentrations found in CF plasma (∼0.3 ng/mL). Next, these cultures were challenged with a standard concentration of LPS, following an ET-model reported before (see ref. [10]). Our data indicated that a concentration of LPS higher than 0.25 ng/mL was enough to induce a refractory state in healthy control cells. In contrast, those doses found in healthy controls and COPD patients (<0.25 ng/mL) did not provoke an ET. In addition, the “pre-treatment” with sera from CF patients but not controls provokes a significant down-regulation of the inflammatory responses after LPS-challenge (**Fig. 4A–4C**). Collectively, these results suggest that the circulating LPS may cause the unresponsiveness state of M
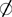
s in CF patients.

As we referred above, endotoxemia has been described in other pathologies, and has been frequently associated to bacteremia [27]. However, circulating infection rarely has been reported in CF pathology. In line with these data, we were unable to detect bacterial DNA in the plasma of our cohort of CF patients. This apparent discordance, on one hand the presence of LPS and, on the other hand, the absence of circulating bacteria in CF patients, could be explained by the high antibodies titres found in CF serum, especially against to the main colonizing bacteria, *Pseudomonas aeruginosa* [28]. In addition, other authors have reported significant amounts of soluble LPS released during bacteria elimination [29], [30]. This endotoxin, like other bacterial detritus, can be absorbed in part from the intestinal lumen by human enterocytes. After bacterial death, their cellular components might be separate into their elements and LPS could cross the gastrointestinal barrier directly into blood like other food nutrients do.

Despite our assays not having clarified the actual source of the LPS detected in CF patients, we have speculated with two not mutually exclusive hypotheses. First, due to the chronic bacterial colonization CF patients' lungs suffer; endotoxin translocation from that organ could be a potential source of circulating LPS. Note that these patients exhibited an inefficiency of the mucociliary system during the clearance of microorganisms progressing in the lower respiratory track [6], [7]. Second, because intestinal inflammation is also a constant feature in CF-patients [31], and this can induce an increased epithelial permeability [32], the gastrointestinal tract is another putative starting place for LPS in the circulation of CF patients. In this line, studies of CF patients' small intestines have reported an increase in mononuclear cells in the duodenum [33] and the enhancement of luminal albumin, immunoglobulins, eosinophil cationic protein, neutrophil elastase, interleukin-1ß, and interleukin-8 [32] detection. The cause of intestinal inflammation in humans with CF is unknown, but around 40% of CF patients have been reported to have microbial overgrowth in the small intestine [34], [35].

Data presented here seems to convey that circulating LPS could be a marker for endotoxin tolerance grade exhibited by CF patients, and may open new avenues of research in the clinical strategies followed during systemic infection in CF patients. In summary, our findings suggest that circulating endotoxin contributes decisively to the ET status in CF patients. The determination of the precise source of circulating LPS awaits further studies.

## Materials and Methods

### Patients and healthy controls

#### CF patients

We studied 14 non-smoker adults diagnosed with CF on the basis of established criteria (clinical phenotype, sweat testing and CFTR genotyping), who had not used corticosteroids within the three months previous to the study. Exclusion criteria included a history of Chronic Obstructive Pulmonary Disease (COPD), asthma or other active lung disease, mental or physical handicap, or other significant diseases such as diabetes mellitus, congestive heart failure, ischemic or valvular cardiopathy or neuromuscular disease.

None of the subjects had an experienced an exacerbation of respiratory tract infection within the previous four weeks. The following clinical variables were collected on each subject: CFTR-genotype, exacerbations in the last year, microorganisms in sputum, and usual therapy. Standard, calibrated scales and stadiometers were used to determine height, weight and body mass index (BMI). Spirometry (FEV_1_ and FVC) was performed with a MasterScope System (Viasys Healthcare, Würtzburg, Germany) according to American Thoracic Society/European Respiratory Society criteria [36]. Results were expressed as percentage of normal values, using best postbronchodilator measurements. Predicted values were calculated from the equations for adults of the European Community for Steel and Coal [37]. See **table 1**.

**Table 1:** 

Subject	Age, yr	Sex	BMI, Kg/m^2^	CFTR Mutation	FEV1, % predicted	FEV1/FVC, %	Microorganism in sputum	Inhaled Bronchodilators	Recombinant human DNasa	Inhaled Antibiotics
1	23	M	20.2	F508/U	55.5	55.9	*P. aeruginosa*	Formoterol	N	Colimycin
2	39	M	25.1	F508/F508	87.4	81.4	*P. aeruginosa*	Formoterol	2.5 mg/d	Tobramycin
3	30	F	21.8	F508/U	32.8	65.3	*P. aeruginosa*	Salbutamol,Ipratropium	N	Colimycin
4	30	M	25.2	F508/F508	73.8	70.6	*P. aeruginosa*	Salmeterol	N	Tobramycin
5	21	M	23.2	R553/2789	85.2	72.9	*P. aeruginosa/S. aureus*	No	N	Tobramycin
6	29	M	21.8	F508/F508	33.9	48.4	*P. aeruginosa*	Salmeterol, Salbutamol	N	Colimycin
7	68	F	26.3	F508/F508	65.0	70.3	*P. aeruginosa*	Salmeterol	N	No
8	32	M	22.8	F508/F508	79.4	75.1	*P. aeruginosa*	Salbutamol	N	No
9	22	F	23.0	F508/F508	35.9	54.4	*P. aeruginosa/S. aureus*	Formoterol/ Formoterol	2.5 mg/d	Tobramycin
10	26	M	21.1	F508/U	82.4	76.2	*P. aeruginosa/S. aureus/H. influenzae*	No	N	No
11	31	M	22.4	F508/F508	51.2	64.3	*P. aeruginosa*	Salmeterol	N	Tobramycin
12	24	M	21.7	F508/U	83.2	71.7	*S. aureus/H. influenzae*	No	N	No
13	23	F	20.3	F508/F508	43.1	65.9	*P. aeruginosa*	Formoterol	N	Colimycin
14	28	F	23.7	F508/U	39.87	67.0	*P. aeruginosa*	Salmeterol, Salbutamol	N	Tobramycin

#### COPD patients

Eight clinically stable patients with moderate-severe COPD (post-bronchodilator FEV_1_ <80% of predicted and FEV_1_/forced vital capacity (FVC)≤70%) [38] were selected. Exclusion criteria included a history of asthma, other active lung disease, mental or physical handicap, or other significant diseases, such as congestive heart failure, ischemic or valvular cardiopathy or neuromuscular disease. None of the subjects had experienced an exacerbation or respiratory tract infection within the previous four weeks, and none of them showed significant bronchodilator reversibility (either >12% of baseline FEV_1_ or >200 ml). No subject has had oral corticosteroid therapy for at least three months.

#### Septic patients

Eight septic patients were consecutively admitted to the Department of Internal Medicine at the Hospital “La Paz” with microbiologically confirmed bacteremia (positive blood cultures for *Escherichia coli*) secondary to urinary tract infection, who met the diagnostic criteria for sepsis by consensus conference definition [39]. Blood samples were taken within 4–8 h after blood culture collection, when they met the sepsis criteria for the first time. The following exclusion criteria were imposed: malignancy and chronic inflammatory diseases, treatments with steroids or immunosuppressive drugs during the last month, hepatic failure (serum aspartate aminotransferase and/or alanine aminotransferase level>100 IU/L; prothrombin time<60%, total bilirubin level>60 lmol/L), renal insufficiency (plasma creatinine level>200 lmol/L), AIDS, virus B or C hepatitis, gestation, and over 70 years of age.

Additionally, eleven age-matched healthy volunteers without personal history of CF or other significant illness were included as controls. Written informed consent was obtained from all subjects enrolled. This study was approved by the local Ethics Committee (‘La Paz’ Hospital Ethics Committee).

### Abs and reagents

The following antibodies were used: anti-CD3-PE (Becton Dickinson; CA, USA); anti-CD14-APC (Miltenyi Biotec; CA, USA). The medium used for cell culture was Dulbecco's MEM from Invitrogen (Paisley, UK). LPS from *Salmonella abortus* was a kind gift from Dr. Galanos (Max-Planck-Insitut für Immunobiologie, Freiburg, Germany). All other reagents were obtained from Sigma-Aldrich (Saint Louis, MI, USA), unless otherwise stated.

### PBMC isolation and cell culture

Starting from peripheral blood from CF, COPD, septic patients or healthy volunteers, adherent cells were purified following the same protocol employed for buffy coats in previous reports from our laboratory [5], [9], [10], [40]–[42]. Note that plasmas from these patients were collected for other analysis. The composition of the adherent population was analyzed by FACS (∼92% of CD14). Once plated, cells were exposed or not to LPS for various times according to particular experiments. All reagents used for cell culture were endotoxin-free, as assayed with the Limulus Amebocyte test (Cambrex; North Brunswick, NJ, USA).

### Endotoxin Tolerance model with human monocytes

Once seeded, adherent cells were treated or not with indicated concentrations of LPS during the “time of tolerization” (t_tol_ = 8 h). After that, cells were washed three times with PBS and kept in complete medium for different times through the phase called “time of recovery” (t_rec_ = 16 h). Then cells were re-stimulated or not with 10 ng/ml of LPS for 24 hours. Note that this model was established by our group in a previous study [10].

### RNA isolation and cDNA synthesis

Cells were washed once with PBS and their RNA was isolated using the High Pure RNA Isolation Kit from Roche Diagnostics (Mannheim, Germany). cDNA was obtained by reverse transcription of 1 µg of RNA using the High Capacity cDNA Reverse Transcription kit from Applied Biosystems (Foster City, CA, USA).

### mRNA quantification

Gene expression levels were analyzed by real-time quantitative PCR (Q-PCR) using the LightCycler system from Roche Diagnostics and cDNA obtained as described above. Q-PCR was performed using a QuantiMix Easy SYG kit from Biotools (Madrid, Spain) and specific primers. Results were normalized to the expression of the β-actin, and the cDNA copy number of each gene of interest was determined using a seven-point standard curve as we described before [5], [9], [10], [40]–[42].

### Primers

The sequences of oligonucleotides used and their annealing temperatures are:

IL-10: sense 5′-ATG CCC CAA GCT GAG AAC CA-3′, antisense 5′-TCT CAA GGG GCT GGG TCA GC-3′ (58°C);

IL12p40: sense 5′-GAC ATT CAG TGT CAA AGC AGC A-3′, antisense 5′-CCT TGT TGT CCC CTC TGA CTC T-3′ (64°C);

IL23p19: sense 5′-GTT CCC CAT ATC CAG TGT GG-3′, antisense 5′-GAG GCT TGG AAT CTG CTG AG-3′ (60°C);

β-actin; sense 5′-GTG GGG CGC CCC AGG CAC CA-3′, antisense 5′-CTC CTT AAT GTC ACG CAC GAT TTC-3′ (60°C).

All primers were synthesized, desalted, and purified by Bonsai Biotech (Madrid, Spain).

### ELISA for TNFα and IL6

Concentrations of TNF-α in supernatants were determined using the ELISA development kit supplied by PeproTech (Rocky Hill, NJ, USA). IL-6 levels in supernatants were determined with a commercial ELISAs purchased from Bender MedSystem (Burlingame, CA, USA).

### Phagocytosis of bacteria assay

We followed protocols previously described by de las Heras and co-workers [43] or da Silva and co-workers [44].

### Proliferation assay

We followed a protocol previously described by Hernández-Fuentes and co-workers and Adam and co-workers [45], [46].

### Endotoxin quantification

The LPS concentrations were determined in 200 µl of plasma using a kit based on a Limulus amaebocyte extract (LAL kit endpoint-QCL1000, Cambrex BioScience, Walkersville, MD). Determinations were done five times per sample.

### Metagenomic assay

DNA was extracted from 200 µl of plasma using a QIAamp tissue kit (Qiagen, Hilden, Germany), and a universal bacteria set of primers for 16S rRNA (sense 5′-ATT AGA TAC CCT GGT AGT CCA-3′ and antisense 5′-AGG CCC GGG AAC GTA TTC AC-3′) were used yielding an amplicon size of ca. 550 bp. All PCRs were carried out in a final volume of 50 µl containing 100 ng of DNA, 0.5 µM of each primer, 0.2 mM of dNTPs, 100 ng/ml of BSA, 3 mM of MgCl2, and 2 U of FastStart Taq polymerase (Roche Diagnostics, Indianapolis, IN, USA). The thermal cycling conditions used were as follows: an initial DNA denaturation step at 95°C for 7 min, followed by 40 cycles of denaturation at 95°C for 30 s, primer annealing at 52°C for 45 s, and a final extension at 72°C for 45 s. Positive amplicons were visualized in both 0.8% agarose gels and separated in vertical electrophoresis polyacrylamide gels (8%) at 60°C; the urea-formamide denaturating gel gradient (33–43%) was submitted to 130 V during 330 min. Gels were visualized with ethidium bromide.

### Data analysis

The number of patients or experiments analyzed is indicated in each figure. In the case of in vitro assays, data were collected from a minimum of three experiments to calculate the mean ± SD and the statistical significance was calculated using the unpaired Student's test and differences were considered significant at *p* values<0.05 using Prism 5.0 software (GraphPad, San Diego, CA, USA). Also ANOVA analysis following of a Turkey test was performed.
